# A comprehensive narrative review exploring the current landscape of digital complete denture technology and advancements

**DOI:** 10.1016/j.heliyon.2025.e41870

**Published:** 2025-01-10

**Authors:** Chan Park

**Affiliations:** Department of Prosthodontics, School of Dentistry, Chonnam National University, Gwangju, Republic of Korea

**Keywords:** Digital denture, CAD/CAM, 3D printing

## Abstract

This review article comprehensively discusses the various treatment protocols and fabrication methods for complete digital dentures and focuses on systematic classification of the techniques introduced in the literature. The treatment protocols are based on conventional procedures, highlighting the areas where digital technologies can be utilized and their advantages. Particularly, it emphasizes the benefits of overcoming time and spatial limitations, along with advantages of permanent data storage. The limitations of digital technologies and potential solutions are also addressed. Current methods and alternatives for patient information acquisition, a common concern among many dentists, are presented. The fabrication methods are categorized into two main manufacturing techniques: milling and three-dimensional (3D) printing. Under the premise of advanced milling technology and limited printing capabilities, their advantages, disadvantages, and applications are compared. Novel hybrid technologies combining both methods are introduced, including challenging aspects of metal framework integration. By examining the complete digital denture fabrication and treatment process from start to finish, this article aims to discuss the present and future of digitally integrated treatment methods.

## 1 Introduction

The scientific approach to dentistry, based on evidence, began with the rehabilitation of complete dentures and the consideration of how to reconstruct the entire arch in edentulous patients. Various schools of thought and theories related to occlusion have emerged, and well-known philosophies such as the Gnathology and PMS schools have attempted to apply the theories of natural tooth treatment to the arrangement of teeth in complete dentures [[1], [2], [3]]. Various occlusal concepts suitable for complete dentures, such as bilateral balanced occlusion and lingualized occlusion, were proposed and have been employed until the present day. The process of gathering patient information, such as impression taking and facial analysis, and the subsequent use of recording bases and wax rims to determine vertical dimensions and centric relations, has continuously evolved based on experience and science [4,5]. Remarkably, even in modern dentistry, the complete denture treatment process remains largely unchanged from the past, and the current digitally-based denture fabrication process is merely a methodological variation of the analog approach [6,7].

The traditional complete denture fabrication process involves a complex series of steps. This process requires the skill of an experienced dentist to gather patient information, but the actual fabrication of the denture itself also demands a comparable level of expertise and time [8]. Achieving an ideal tooth arrangement on a precise articulator setting, satisfying both aesthetic and functional needs, is no easy task. Even after completing the denture on the articulator, the process of curing the resin requires various skills to minimize errors [9]. Furthermore, the development of materials that can withstand long periods in the patient's oral cavity and the possibility of future repairs necessitate immense patience and time from both the patient and the practitioner. In particular, for edentulous patients, the several months required for fabrication can be an arduous period. Therefore, the need for digital methods in denture treatment has evolved through several pathways to optimize the process and eliminate time constraints [10].

The integration of digital technologies in dental prosthesis fabrication, known as computer-aided-design/computer-aided manufacturing (CAD/CAM) technology, is believed to have begun in the 1970s with Professor Francois Duret of France and the development of the Chairside Economical Restoration of Aesthetic Ceramics (CEREC) system by Dentsply Sirona in the 1980s [11,12]. These technologies were initially used to produce simple inlays or single crowns, but as the technology progressed, attempts were made to fabricate more complex prostheses. The use of digital dentures in the literature is reported to have started around 1990 [13]. In the early 1990s, the first attempts to fabricate complete dentures using CAD/CAM technology utilizing 3D laser scanning and milling techniques were reported [13,14]. In the 2000s, advancements in intraoral scanning and three-dimensional (3D) printing technologies led to the development of several digital denture systems [15], such as the Dentca CAD/CAM denture system [16] and the AvaDent digital denture system [17]. In the 2010s, the introduction of new materials and improvements in digital workflows further expanded the application of digital technologies in complete denture fabrication [18]. More recently, the integration of artificial intelligence (AI) and machine learning in digital dentistry has created new possibilities for automating and optimizing the denture design and fabrication process [19,20].

As digital denture treatment continues to advance, the fabrication process has become simpler, while the manufacturing has become more sophisticated. The current level of digital denture treatment is such that if the dentist accurately captures patient information, the fabrication stage can already achieve a high degree of completion and is well-regarded [21]. However, this is not the end. The process of acquiring patient information using digital equipment also requires considerable skill, and if traditional impression methods are used, an understanding of how this information is converted into digital data is necessary. Consideration of the final denture materials is also essential, and based on this, the dentist must understand the prognosis of the treatment and take responsibility for continuous maintenance and management. In this regard, it is crucial to divide digital denture treatment into the clinical stage of acquiring patient information and the dental lab stage of fabricating the denture and to understand which aspects can be or have been digitized at each stage as well as the limitations of digitization. In this paper, we aim to examine the overall protocol of digital dentures, discuss the considerations and limitations at each stage, and explore potential solutions and future directions. By analyzing the various processes involved, we endeavor to provide a comprehensive understanding of the current state of digital denture technology and its practical applications, as well as identify areas for further improvement and advancement.

This paper is a narrative review, describing the clinical process, laboratory process, considerations and limitations, and methods to overcome the challenges of digital dentures. The clinical process first explains the advantages of digital patient data, followed by conventional steps such as impression taking, vertical dimension and centric relation recording, and anterior tooth positioning. The laboratory process begins with the CAD procedure, while the CAM process is broadly divided into milling and 3D printing. Subsequently, the paper analyzes the limitations of digital dentures presented to date, explores ways to overcome these limitations, and concludes. A literature search was conducted on PubMed using the keywords "denture," "complete denture," and "digital denture." Papers published since the 1960s were selected for general denture theory and evidence, while those published since the 1990s were chosen for digital denture content. Ninety four papers were selected as references, focusing on content that aligns with the author's opinions.

## 2 Method

This study focused on two primary keywords: "complete denture" and "digital denture." I conducted searches across four electronic databases: PubMed/MEDLINE, Scopus, Web of Science, and Embase. We strictly adhered to the PRISMA guidelines throughout the review process. Different search periods were set for each keyword. For "complete denture," we limited the search from January 1960 to December 1990 to capture content aligning with conventional denture theory. For "digital denture," the search spanned from January 1960 to January 2024. However, as the concept of digital dentistry was primarily established post-1990, there was minimal temporal overlap between the two databases.

Boolean operators "AND" and "OR" were employed to construct search strings, such as (complete denture OR full denture) AND (digital OR CAD/CAM). This study included clinical studies on complete digital dentures, including in vitro, in vivo, case reports, and dental technique studies. The exclusion criteria were as follows. 1) Non-English literature. 2) Studies utilizing finite element analysis, as some discrepancies were observed between these and clinical outcomes. After removing duplicates, 94 articles were ultimately included in this review.

The data from the included papers were categorized and synthesized according to the definition of digital dentures, their advantages, patient data collection methods, design approaches, fabrication techniques, and pertinent discussion points. Considering the heterogeneity of the included studies, a meta-analysis was not performed. Instead, we conducted a narrative synthesis, focusing on various aspects relevant to digital dentures.

To evaluate the study quality, assessment criteria developed by the author were employed. These criteria considered the appropriateness of the research design, adequacy of the sample size, reliability of the data collection methods, and clinical relevance of the findings. Each paper was evaluated according to these criteria. the outcomes of the assessment were incorporated into the results interpretation process.

A primary limitation of this review is the potential for increased subjectivity due to the single-author review process. To minimize this, we established clear selection criteria and followed a systematic review process, guided by the PRISMA framework. Additionally, the rapid advancement of digital denture technology means that the content of this review may soon become outdated.

A narrative review approach was chosen because digital denture technology is an evolving field with a notable gap between academic research and practical applications. While we followed PRISMA guidelines for transparency and reproducibility, the rigid methodology of a systematic review might limit the comprehensive exploration of various aspects. Therefore, this study explored the current technological trends and clinical applications more flexibly, while also ensuring that our analyses were comprehensive, balancing academic findings with practical applications.

## 3 Denture treatment protocol

### 3.1 Time-space transcending benefits of digital patient data management

Digital patient data management in denture treatment has evolved significantly with the advancement of AI technology [22]. The comprehensive patient information captured includes detailed records of the oral cavity in both two-dimensional (2D) and three-dimensional (3D) forms, enabling instantaneous visualization and analysis. These data demonstrate particular value in tracking individual changes over time. For example, if the dentition state of a healthy patient is stored in advance, these data can be used for tooth arrangement when denture treatment is needed later. One of the most significant advantages is that these data can be stored permanently and accessed anytime, anywhere, by anyone. In other words, this enables work that transcends time and space. For instance, information acquired in one region can instantly be transferred to the opposite side of the globe, allowing the work to be performed even during sleeping hours in that region [23]. Of course, various treatment protocols will be necessary after fully recognizing and discussing related issues such as security concerns, privacy infringement, and legal differences between countries [24].

### 3.2 Patient information acquisition

#### 3.2.1 Impression taking

Perhaps the most important aspect that many dentists consider when treating with complete dentures is how well the impressions are taken. Compared with taking impressions of the hard tissues of teeth, taking impressions of the soft tissues of the gingiva or alveolar ridge is a technique-sensitive task often referred to as "impression making." [25] The results obtained from this process are undeniably the key factors that determine the quality of the denture. In other words, before discussing digital impression taking, it is crucial to recognize that impression taking is of utmost importance in denture treatment, regardless of the method used.

The digital method of edentulous impression taking involves scanning with an intraoral scanner (IOS). To achieve successful edentulous impression taking, it is necessary to understand the principles of an IOS. As an IOS is used inside the mouth, it captures only very small areas; therefore, to receive data for the entire arch, numerous captured images must be stitched together [26]. During this process, stitching relies on the continuity of the same scene recognized by the IOS. However, in the case of edentulism, many parts of the oral cavity are similar in appearance, making it difficult for the stitching algorithm to proceed. This can result in incomplete scanning or sometimes distorted intraoral scans due to incorrect stitching [27]. While the current scanning technology is known to have few problems with scans for fixed prosthesis restoration [28], removable prosthesis restoration remains relatively difficult [29].

However, there is room for a different perspective regarding the difficulty of edentulous scanning compared to the results. Removable prostheses do not require the same level of precision with fixed prostheses. Some studies have suggested that impression discrepancies of approximately 2 mm are acceptable in denture treatment [30], while others have reported that a compression of approximately 500 μm due to impression pressure does not cause a significant difference in sensory reception [31]. Therefore, the role of the dentist is to incorporate digitized methods while taking advantage of these aspects.

Another challenge in edentulous scanning is the morphological aspect of the IOS, which makes it difficult to scan areas where the IOS cannot reach, such as the retromolar pad [32]. Moreover, since it is a method of capturing borders that does not require pressure, it is difficult to obtain sealing, which is also a limitation [33]. The difficulty in morphological access can be somewhat overcome by the practitioner's technical proficiency, but there is currently no clear solution for border sealing, and a different type of scanner needs to be developed. However, even if the border sealing is not perfect, denture treatment outcomes are reported to be not poor if there is no severe alveolar bone resorption or if overall excellent impression taking and occlusal relationship setting are achieved [34].

If the practitioner determines that edentulous scanning is impossible for any reason (lack of skill or various patient factors), the digital denture treatment protocol can be continued with slight modifications. Namely, after completing the impression taking using the conventional method, this impression body is scanned. This method is performed outside the mouth, making scanning much easier, and has the advantage of relatively accurately recognizing the anatomical structures recorded in the impression. In addition to IOSs, this can also be performed with model scanners. Although IOSs are excellent, model scanners show a better precision; thus, there is no problem in digitizing analog data using this method [35]. Using these two methods interchangeably according to the practitioner's convenience will be highly advantageous in accommodating various advantages.

Therefore, we propose the following edentulous patient intraoral scanning protocol for digital complete dentures. First, attempt edentulous scanning using a scanner. If the desired scanning results are obtained within a short time, it is safe to proceed to the next stage. If scanning fails despite multiple attempts, proceed to the next stage by taking impressions using the traditional method and scanning the impression body. If the scanning is relatively successful but the quality of the results is questionable, the scanning data are used only to fabricate custom trays. If custom trays are fabricated using 3D printing or other methods, they can be used as relatively satisfactory trays for conventional impressions. Of course, this process has significant time and cost savings.

#### 3.2.2 Vertical dimension and centric relation taking

Many dentists likely consider this process to be even more difficult than impression taking and approach it accordingly. In particular, in the case of edentulism in which teeth are missing, capturing the patient's vertical dimension (VD) and centric relation (CR) is a very challenging treatment process. Determining the VD requires a comprehensive examination of factors, such as the patient's facial appearance and pronunciation, while capturing the CR involves observing the position of the temporomandibular joint (TMJ) beyond the oral cavity and considering functional aspects [36,37]. If the patient has been edentulous for a long time, acquiring this information becomes even more difficult, and a process may be necessary where the dentist's experience rather than the patient's sensation is used to find and establish the appropriate position [38]. The goal of the digital denture treatment process is to shorten the time as much as possible, but the process of quickly finding such difficult information is a very challenging obstacle to overcome in achieving that goal.

To date, no digital methods for capturing the VD and CR have been reported. The current state involves using traditional methods such as gothic arch tracers or capturing the information using the conventional method based on scanned data to create a recording base. Many dentists have attempted to overcome this with several devices, and recently, efforts are being made to acquire denture information using cone-beam computed tomography (CBCT), which contains considerable information [39]; however, it is still in the early stages and its practical application remains uncertain. If there is a way to capture the natural bite posture with CBCT and integrate it with intraoral data, it would be a truly digital method of acquiring the VD and CR [40]. Alternatively, an approach could be to collect multiple data points and use AI to extract approximate VD and CR information.

Companies offering various digital denture solutions present methods of taking impressions using trays, cutting or separating them, attaching devices such as gothic arches, and capturing the VD and CR [41]. If this process is performed simultaneously with impression taking, considerable expertise is necessary. A more comfortable method for dentists is to separate the scanning process, create a recording base and wax rim based on the scanned data, and then perform the VD and CR taking process [42].

**Fig. 1:**
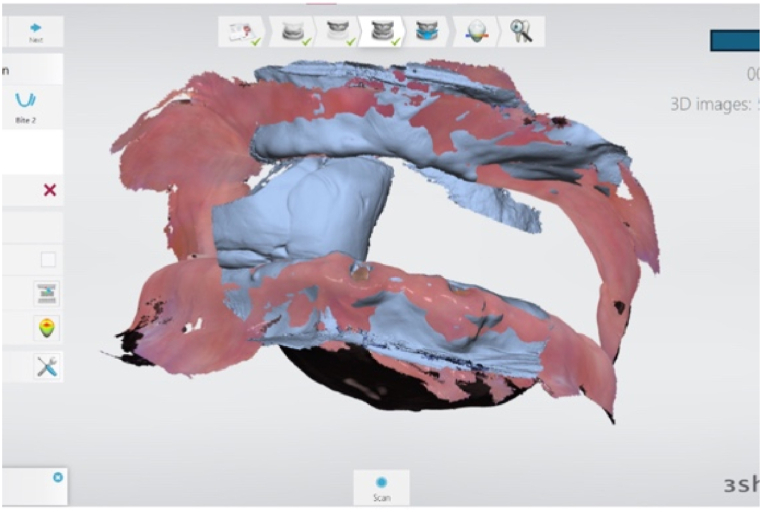
Acquiring VD and CR records using an intraoral scanner while exposing the anterior region.

Another option is to roughly perform the VD and CR taking process through scanning and then create a more manipulable recording base and rim based on those data. In other words, after scanning the maxillary and mandibular edentulous areas, when capturing the occlusal relationship, a dense material such as putty is used only in the posterior region to create stop points (Fig. 1). Before creating these stop points, it is essential to train the patient to bite in the CR position with the TMJ using the bimanual method and to assess the facial appearance to ensure they stop at the appropriate position. If the occlusion is recorded with an IOS in this stopped state, the occlusion will be captured based on the shape of the exposed edentulous area in the anterior region. If the previous stop point is judged to be the perfect VD and CR position, then excellent patient information acquisition has been achieved very easily. If there is a slight error in this process, a recording base and rim can be fabricated based on these data, and the relevant information can be captured again in the patient's mouth. The advantage of these roughly captured data is a shortened time to capture VD and CR later.

To summarize the VD and CR taking process again, since this process cannot be completely digitized even when attempting digital dentures, an approach that utilizes existing methods should be taken. Traditional recording bases and wax rims can be used, or gothic arch tracers can be employed in addition. There is also a method of taking the VD and CR simultaneously with impression taking using the closed-mouth technique. Specially fabricated trays that are separated or cut may be difficult to use; therefore, a cautious approach is necessary. The author recommends the so-called open-tray method in which the anterior edentulous area is opened, bite registration material is inserted only in the posterior region, and the occlusion is captured with an IOS as a protocol for future digital denture treatment. Furthermore, it is hoped that more convenient technologies will be developed through the active utilization of CBCT and other devices to capture multiple data points.

#### 3.2.3 Anterior tooth position

The basis for tooth arrangement in complete dentures is generally set to pursue aesthetics in the anterior region and functionality in the posterior region [43,44]. The arrangement of posterior teeth, which emphasizes functionality, can be achieved with well-aligned patient data (facial scans, intraoral scans, CBCT, etc.) and the corresponding movements of a virtual articulator. If jaw motion tracking data can be added, it is possible to arrange perfectly functional teeth [45]. However, for the anterior region, aesthetic tooth arrangement is of utmost importance, even if some functionality is sacrificed [44]. Therefore, the dentist's judgment and confirmation of the aesthetic tooth position are essential for a lifelike anterior arrangement. The only way to confirm the position of the anterior teeth in complete denture treatment is to predict it using the wax rim on the recording base [46]. If one were to set the position of the anterior teeth without using a wax rim, except for the method described in Fig. 1 or analog methods, it would be a very challenging task.

A method of setting the position of the anterior teeth without relying on wax rim information is to predict it based on anatomical knowledge [[47], [48], [49]]. For example, this method utilizes the information that the center of the incisive papilla is originally located 8–10 mm behind the position of the central incisor. The relative position can also be determined based on the location of the remaining teeth. If the patient is edentulous in the maxilla and has anterior teeth in the mandible, the position of the maxillary teeth relative to the mandibular teeth can be determined after setting the vertical dimension. When determining this position, the dentist's experience, the patient's sensation, or photographs from when the patient was younger can be used as references. However, the abovementioned methods are based on the premise of not fabricating a wax rim. Most dentists intuitively do not use such methods when they cannot directly confirm the position of the anterior teeth. The digital denture protocol aims to reduce the number of treatment visits, which creates a dilemma.

**Fig. 2:**
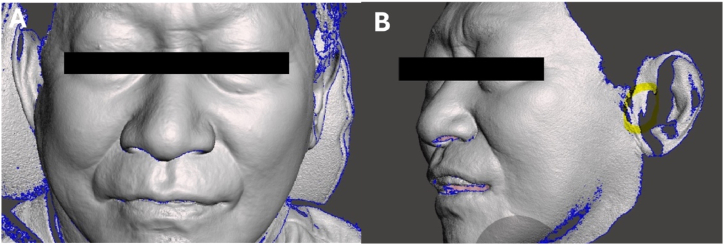
Facial scanning aspect. Fig. 2A) Frontal view with denture, Fig. 2B) Lateral view with denture. Facial scanning can be used to evaluate the appropriateness of the anterior tooth position by detecting changes in the facial appearance.

Therefore, this paper proposes a new method. If a facial scanner is used among digital devices, the facial appearance can be confirmed. Assuming the treatment of a maxillary edentulous patient, first, the patient is instructed to bite naturally in the position where the lips gently touch in the most upright posture(Fig. 2). If a facial scan is taken, the facial profile on the lateral view can be observed, and the lip position at this time can be expected to be approximately at the position of the current alveolar bone. In almost all patients, the lateral view shows an unaesthetic state with retruded lips. On this display, the position of the maxillary anterior lip is predicted to be protruded by approximately 1–2 mm beyond the mandibular anterior position. Therefore, based on this, how far labially the maxillary anterior teeth should be positioned from the uppermost position of the current alveolar ridge can be calculated. Of course, since this method is only an estimate, a try-in denture should be fabricated to actually confirm it and obtain the patient's consent.

If a definite method using a wax rim is desired, digital technology focuses on quickly fabricating the rim. In other words, after acquiring maxillary and mandibular edentulous scans recording bases are 3D printed directly using the scanned data, and wax is applied at appropriate positions. This method is not significantly different from the conventional method, but with current technology, scanning and printing can be completed within an hour, allowing for same-day treatment without additional patient visits [50].

To summarize the anterior tooth position again, the position of the maxillary anterior teeth is determined by predicting the aesthetic position of the lateral facial profile based on anatomical indicators of the edentulous area and facial scanning. A rough try-in denture is fabricated at the determined position and re-evaluated to obtain the patient's consent. If it is necessary to identify the anterior tooth position through a recording base and wax rim, a method of quickly printing the base using the scanned edentulous data can be chosen, which, although not significantly different from the conventional method, enables faster treatment. While not yet practical, it is expected that the use of CBCT, which contains 3D hard and soft tissue data, will enable easier and more predictable anterior tooth position setting in the future.

## 4 Digital manufacturing process

### 4.1 Digital denture design: from data integration to virtual articulation

#### 4.1.1 Superimposition of patient data

Once all patient information has been acquired, the next step is the domain of dental technology. At this point, it is important to integrate all of the patient information based on accurate coordinate values [51]. In particular, for complete dentures, since the work cannot be performed on a simple hinge articulator, the oral data must be set on a virtual articulator [52]. There are several methods to set patient data on a virtual articulator. The first is a completely analog method in which the patient's stone model is mounted on an articulator; then, all objects (materials) including the articulator are scanned with a model scanner. This method is identical to the fully analog approach, and only the processes after CAD are digitized. The second method involves merging facial scan or CBCT data with intraoral scan data and applying a virtual articulator based on the recorded TMJ position [53]. If a jaw motion tracking device is used, the patient's movements can also be applied as values to the virtual articulator, resulting in data that incorporate more complete patient motion. Finally, there is a method that modifies the previously used facebow. Using a special facebow device that can input digital values, the facebow is set on the patient in the traditional manner, and only the resulting values are applied in CAD, achieving the effect of mounting on an articulator based on the facebow in a virtual space. This has currently been introduced as a product of a specific company [54], and this method can be used by existing dentists without much unfamiliarity by combining digital and analog methods. The use of a virtual articulator not only eliminates the cumbersome gypsum mounting process of the past but also avoids the errors that occur during this process, making it a very efficient digital protocol. In addition, since various articulators can be used, simple or very sophisticated articulators can be employed as needed [55].

#### 4.1.2 Anatomic landmark setting

**Fig. 3:**
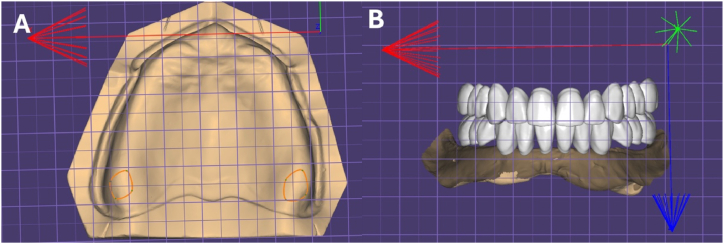
Diagnosis of edentulous arch by dental CAD software. The same analyses that were performed on traditional plaster models can also be carried out on CAD models, and these digital analyses can demonstrate easier and more accurate quantification. Fig. 3A) Edentulous maxillary model placed on graph paper, allowing for easier assessment of lengths and sizes at various positions. Fig. 3B) Teeth arrangement placed on graph paper, enabling easy distinction of the relative sizes and lengths of the teeth.

Once the superimposition of all patient data is complete, the next important process is to set anatomical guidelines for tooth arrangement. This part relies heavily on knowledge of human anatomy, and the principles and knowledge of dentistry are crucial before discussing the methods. Most CAD software sets the tooth arrangement based on these settings [56,57]; thus, more accurate position setting is necessary to avoid too many modifications later. The incisive papilla, hamular notch, retromolar pad, and approximate canine positions are used. The author recommends that the dentist identify these positions in the patient's oral cavity during scanning, mark them with an indelible pencil, and then scan them [58]. Most recent scanners have color information; therefore, they can be easily distinguished in CAD. Alternatively, if files of various shapes of reference points used for model analysis in analog methods, such as symmetrical grid paper or compasses, are created and applied to the patient's scanning data, it will be even more useful for model analysis(Fig. 3).

#### 4.1.3 Artificial tooth arrangement

Arranging teeth in CAD is not significantly different from the analog method. Artificial teeth of appropriate sizes are selected and arranged considering the aesthetics of the anterior region and the occlusion between the maxilla and mandible [43]. The only difference is the use of a mouse and keyboard on a 2D computer screen. However, the aforementioned adjustment on a 2D screen is the main reason it is difficult for dentists or dental technicians to intuitively confirm the results. Therefore, when arranging teeth through the digital process, it is necessary to proceed while considering approaches for other conveniences rather than just simplifying the difficulty of the existing process.

Most software suggests artificial teeth of appropriate sizes based on the previously marked landmarks. If the landmarks are accurate, the selected artificial tooth sizes will likely be desirable. However, the final judgment must be made by confirming the aesthetics through facial scans and other means [59]. In particular, for mandibular edentulous patients, for stability, the posterior cusps should be located within the triangle connecting the canine area to the left and right retromolar pads, depending on the position or condition of the mandibular posterior alveolar ridge, and the size of the teeth should be selected so that the second molar is not positioned on the pad slope [43].

Subsequent tooth arrangement is performed similarly to the analog method of moving individual teeth on a wax denture. Of course, there are convenient tools such as automatic cusp deletion according to the movements of the virtual articulator or setting and moving teeth in groups. However, complete denture tooth arrangement still requires highly skilled techniques and time. It is expected that automated tooth arrangement technologies using AI will be developed in the future, but since each complete denture is created under very different conditions, it will likely take considerable time until a large volume of data are collected. In summary, tooth arrangement is performed in a digital environment similar to the analog process and thus requires significant time and effort. Moreover, since it involves handling three-dimensional data on a two-dimensional screen, skilled experience and proficiency are important. To overcome this, the collection of a large amount of complete denture data is emphasized for developing various methods.

#### 4.1.4 CAD finish process

Recent denture software provides many tools for imparting aesthetics. For example, to create a natural gingival appearance, patterns are applied to the denture base, or scallops between the teeth and denture base can be decorated in several ways. This may vary depending on the preferences of the practitioner or patient, but it is necessary to consider how the denture will be fabricated when making the selection. In addition, the finishing process is a confirmation step to ensure that there are no problems with each part of the final denture [60]. If certain areas are too thick or thin, abnormalities may occur in the final product; therefore, this must be evaluated. Finally, smoothing is performed to soften any sharp or irregular appearances that may cause discomfort to the patient. All of these processes should be selected based on the final production technology. In other words, a design that is advantageous or suitable for each technology, whether it is a milling technique that cuts discs or a printing technique that attaches and photopolymerizes liquid resin, should be chosen. A high-quality design ultimately means that the final product is successfully completed.

### 4.2 CAM process

#### 4.2.1 Consideration of a one-piece or two-piece denture

The design of digital dentures is largely divided into two categories: whether the teeth and base are separated or not. Since the teeth and base have different colors, the basic method is to fabricate them separately. However, to eliminate the errors that occur when combining the teeth and base and for convenience of fabrication, a one-piece method is sometimes used. Try-in dentures for confirming tooth arrangement or occlusion are usually fabricated as one-piece dentures, and if a permanent denture is to be fabricated as a one-piece denture, the entire denture is constructed in tooth color; then, the base area is additionally colored with pink resin.

The biggest advantage of a one-piece denture is that there is no error in occlusion at all because there is no combining process involving the teeth and base. This technique also has the benefit of cost and time savings since it can be completed with a single milling machine or printer in the CAM process. However, additional manual work is required for aesthetics, and the base and teeth cannot simultaneously satisfy the respective physical properties required, which is a current limitation for use as a permanent denture. On the other hand, two-piece dentures exhibit suitable aesthetics and physical properties similar to permanent dentures. However, to produce a high-quality denture, precise techniques are required when combining the base and teeth, and there is a slightly more consumption of equipment and materials.

Printing materials have recently been introduced that change the color of the base to pink after denture printing [61], and milling discs with mixed teeth and base blocks have been released, enabling one-body dentures where the teeth and base are each shaped [62]. In summary, one-piece dentures with economic and convenience advantages are suitable for use as try-in dentures or temporary dentures, while two-piece dentures have several advantages for high-quality fabrication. These technologies are converging and evolving further.

#### 4.2.2 Fabrication method

##### 4.2.2.1 Milling process (subtractive method)

The CAM process using milling machines is a common method of producing prostheses in dentistry today [63]. The burs that move based on two or three axes in 3D space operate on the same principle as a dentist preparing a tooth. Recently, 5-axis milling machines have been developed, where not only the tool but also the disc or block material moves, enabling more precise prosthesis fabrication. In particular, the use of various bur shapes has made it possible to reproduce the border or internal irregularity of dentures, and convenience features such as automatic block change systems and discs containing two or more materials have been developed [63].

The subtractive manufacturing method of producing the final material has the advantage of superior accuracy compared with additive manufacturing because no post-processing is required [64]. The final physical properties are also clear and predictable. In the case of digital dentures, most are made of polymethyl methacrylate (PMMA), and the advantages of various PMMA disc materials developed long ago can be directly utilized. Relatively recently developed 3D printing materials with uncertain long-term follow-up results are expected to yield more stable and better outcomes [65]. In terms of aesthetics, PMMA exhibits a level of aesthetics similar to the previously used pink resin, and the gradiented teeth also show much better shades than single-color printed teeth [66].

However, milling has less freedom of shape than printing; thus, it is necessary to consider the limitations of tool angles and diameters when proceeding with CAD design and the CAM process. In other words, rather than reproducing the appearance of edentulism as it is, a design suitable for milling should be considered. This means that the results may not match 100 % with the intraoral scan data. However, it is known that this level of accuracy does not have a significant clinical impact in digital denture cases [67]. The tolerance allowed for the fit on the edentulous area, where some leeway is acceptable, can be a key to overcoming the limitations of milling [68]. These limitations are also being gradually overcome through the continuous development of milling machines. The author currently considers digital dentures completed through one-body milling using discs with both the tooth and gingival parts to be the most high-quality dentures in many aspects.

##### 4.2.2.2 3D printing

Additive manufacturing, which is the method of building objects by stacking materials layer by layer, was originally limited to polymers and referred to as 3D printing; however, this term is currently used in all fields to describe this type of layered fabrication method [69]. 3D printing is not only much more economical than milling because there is no material to be cut away but also has no limitations in shape due to the limitations of the cutting tool, allowing for the production of very freely shaped products. This idea, developed around 1980,has been treated as a groundbreaking solution to overcome both the manufacturing technology and economics of the past and has been developed and used in various parts of the dental industry as well as general manufacturing industries [70].

In dentistry, 3D printing is considered to be a suitable production method for dentures, which are the main area of edentulous surfaces, especially due to of the absence of shape limitations with this technique. Although much improved now, many milling machines still have angular limitations of the bur during cutting, making it impossible to properly fabricate denture areas with angles outside the limited range. Even if this was possible, it requires consideration in the CAM software strategy regarding how to proceed with the milling.

However, in 3D printing, the installation of supports on the product during output must be considered. Many supports are essential for stable printing output [71,72]. Although the supports are removed, the slight traces of supports that remain definitely require manual removal and polishing. At this time, incorrect manual work can lead to errors in precision; therefore, it is necessary to consider in advance where and how to attach the supports. Recent printing technologies have minimized manual work through the development of various software technologies for easy support removal, and it is expected that automated support location search functions using AI will be added in the future.

**Fig. 4:**
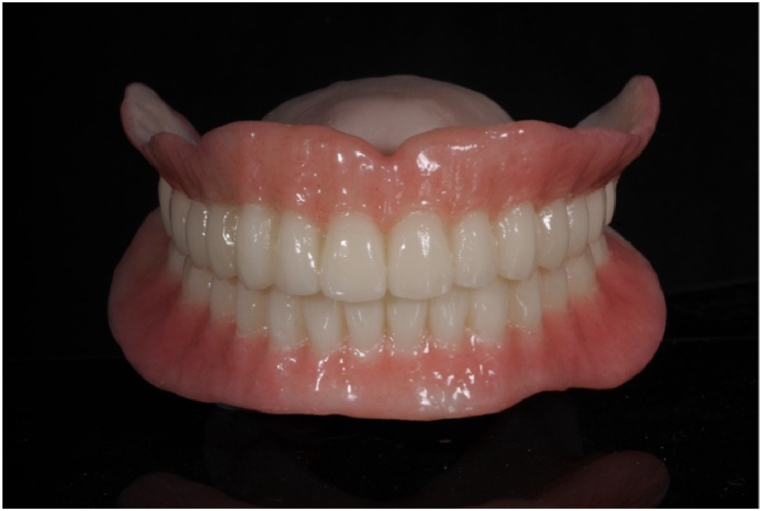
Monolithic digital denture with pink aesthetic. The process can be carried out by printing the crown using a resin material suitable for crown fabrication in a single step, and then adding pink resin to create the gingival areas.

Currently, printing for digital dentures mainly adopts the Stereolithography Apparatus(SLA) method of photopolymerizing photocurable resin using light, and Liquid Crystal Display(LCD) or Direct Light Project(DLP) printers are used depending on the light source [73]. However, since dentures largely consist of two parts, two materials must be used. Many commercially available printers are usually capable of outputting only one material; thus, for final dentures but not try-in dentures, two printers or two outputs are required. Moreover, due to the characteristics of vat photopolymerization printers, it is still difficult to produce aesthetic colors with printing materials that can realize gradiented teeth or various base colors compared to traditional methods. Therefore, if a more aesthetic denture is desired, additional work using various resins, staining, and glazing materials must be performed on the printed digital denture (Fig. 4).

Unlike milled products, printing requires an additional post-processing step after the printing process [74,75]. In other words, excess resin remaining on the surface after printing must be washed, and an additional post-curing process for the uncured resin inside must be performed. Resin washing is done by placing the output in a machine that generates ultrasound or waves along with a cleaning liquid. Isopropyl alcohol (IPA) or special cleaning solutions are used, and the optimal cleaning solution, concentration, and cleaning time depend the printing material [76]. Each process also has various optimized procedures depending on the material and situation; therefore, considering these factors is necessary to produce the exact product originally planned. This is the difference from milling systems that produce relatively consistent quality.

## 5 Limitations of digital methods

Despite the advancements in digital dentistry, the digitalization of removable dentures still faces various challenges compared with fixed prostheses and has not been fully realized [77,78]. These limitations range from difficulties on the clinical side to various issues on the laboratory side [77]. Currently, a combination of conventional analog methods and digital techniques is often employed to overcome these limitations. However, this approach may not fully harness the absolute advantages of digital methods, particularly from the perspective of dental clinicians. Nevertheless, these limitations are gradually being addressed through the development of materials and technologies as well as creative attempts [70]. This section aims to identify the current limitations in the fabrication of digital complete dentures.

### 5.1 Limitations on the clinical side

Perhaps the most challenging aspect of digital complete denture treatment is the intraoral scanning of edentulous arches. The term "impression making" rather than "impression taking" has traditionally been used to describe the process of capturing edentulous impressions, reflecting the need for the clinician's treatment concept to be applied to the variable oral environment [32]. However, when directly using a scanner, the option to control pressure is eliminated, making "impression making" difficult. While there are various reasons for this, the primary purpose of applying selective pressure during impression taking is to achieve retention [47]. Capturing patient information through edentulous scanning without reliably ensuring retention can be a daunting challenge for dentists.

Another difficulty in the scanning process is the limitation of stitching. Current dental scanning technology relies on stitching small scanning sites together based on overlapping scenes [61]. While this method is effective for dentate arches with well-defined shapes, it is relatively more challenging for edentulous areas [79]. As a result, stitching may not proceed properly, or incorrectly recognized stitching can often be observed. Although AI-assisted scanning technologies have significantly improved the techniques for edentulous scanning, they still lag behind those for dentate scanning. Particularly challenging are the posterior regions of the oral cavity (hamular notch, retromolar pad, etc.) that are crucial for complete dentures but difficult for scanners to access. Unfortunately, most scanners cannot be developed specifically for edentulous cases due to the frequency of use, leaving us with the task of creating new solutions to overcome these scanning limitations.

### 5.2 Limitations on the laboratory side

#### 5.2.1 CAD process: 3D intuition, aesthetics, and software specifications

Designing an aesthetic and highly refined digital complete denture still requires a significant amount of time. It demands a different level of data, technology, and insight compared with the automated production of single crowns using AI technology. Therefore, if one desires to digitally produce a complete denture of above-average quality, considerable time and effort are still necessary [80].

The most challenging aspect of the design process is that CAD involves working with 3D objects on a 2D computer monitor. Directly observing and manipulating objects on an articulator allows for more intuitive and quick judgments. However, even when using a virtual articulator with a mouse on a computer, the flat screen makes it very difficult for non-expert dentists or dental technicians to judge distances and positions. Current technology cannot overcome this digital work method; therefore, research on image shifting between 2D and 3D is crucial for smooth workflows [81].

In addition to the difficulty of reconstructing the entire arch in CAD, dental CAD software for removable prostheses is relatively less developed than that for fixed restorations, as mentioned in the scanning technology section. From the perspective of consumers who desire shorter work times than analog methods, this can be a reason to hesitate in adopting digital dentures. However, this software is gradually improving and has now reached a significant level. It is expected that software updates enabling the easy digital replication of analog methods will be available in the near future.

Finally, it is relatively more challenging to achieve the aesthetics of hand-carved gingival wax using a mouse, hindering the production of aesthetic dentures. This aspect is also related to the CAM materials discussed in the next section. Currently, the aesthetics of digital dentures are generally considered inferior to those of conventional dentures. Alternatively, attempting to achieve such aesthetics may require even more time than traditional analog dentures. However, this issue is also expected to be gradually resolved, with various options for aesthetics being introduced in connection with materials and equipment.

#### 5.2.2 Limitations on the laboratory side (CAM): Material properties, aesthetics, equipment purchase, and specifications

##### 5.2.2.1 Material properties

Materials for digital complete dentures have shown remarkable progress in recent years, with some results demonstrating superior physical properties compared to conventional materials [82,83]. Replicating porcelain teeth through milling or printing remains extremely challenging, which consequently limits fabrication to PMMA-based dentures [84].

However, despite promising results in laboratory settings, the wear resistance of digital materials in clinical applications often reveals significant wear patterns. This limitation is particularly evident in 3D printed dentures, which has led to their restricted use as provisional prostheses only [84]. Fortunately, milling blocks have undergone significant advancements and now exhibit strengths nearly equivalent to those of conventional materials [85]. However, milling dentures takes considerably more time than printing, and milling machines are generally much more expensive than 3D printers.

##### 5.2.2.2 Aesthetic limitations of materials

The limitations of materials also impact aesthetic aspects [86]. Most printing methods used for prosthesis fabrication in dentistry involve vat photopolymerization, meaning that digital dentures are made from a single liquid material in a bath. Liquid printing materials cannot reproduce detailed gingival colors or gradated teeth. In other words, manual work is still required after fabrication to achieve aesthetic digital dentures [87]. Milling blocks are relatively more advanced than printing materials, and the development of materials for fixed prostheses has enabled the production of teeth with significant aesthetic quality [88]. However, the pink PMMA blocks used for the denture base still fall short of the naturalness achieved by conventional materials [89]. Aesthetic dentures continue to require manual work, and completely new concepts or the introduction of different printing methods are necessary, especially in the field of printing.

##### 5.2.2.3 Financial and technical challenges in CAM implementation

CAM equipment is also driven by software; thus, the capabilities and specifications of the software significantly influence the quality of the dentures [90]. CAM software for milling machines calculates tool paths and performs other functions, while 3D printing software slices models into precise thicknesses [91]. They can also detect and control issues during the fabrication process. It is crucial to assess how well the software performs functions suitable for digital denture fabrication [92].

The precision and durability of the hardware also affect denture production. Whether it is a milling machine with precise and diverse milling tools or a 3D printer equipped with a high-resolution light source, these factors are essential for denture quality [93]. Moreover, maintaining consistent quality across multiple denture fabrications is the most important clinical criterion [94]. However, as the frequency of machine use increases, many problems such as tool wear, light source weakening, and clearance issues in other parts can occur. These limitations become more pronounced when milling metals or employing metal powder printing processes, where dimensional discrepancies tend to be more significant [95]. Furthermore, the fabrication of metal frameworks necessitates an integration process with the teeth or denture base, which typically requires physical models or analog workflows. While there are limited studies documenting the incorporation of metal frameworks in digital denture fabrication [96], several technical challenges must be addressed before widespread clinical implementation becomes feasible. Therefore, continuous monitoring is necessary to minimize these errors, and calibration may be required to reestablish reference points when needed.

The considerations for the aforementioned equipment ultimately lead to financial expenditures, which can be an obstacle to the digital transition in denture treatment. However, these drawbacks are gradually being overcome through technological advancements. The development of practical milling machines and 3D printers specialized for digital dentures will enable better digitalized treatments.

## 6 Discussion: collaboration and concerns between conventional methods and digital techniques

As mentioned earlier, digital dentures have not completely replaced conventional dentures as a treatment protocol due to various reasons and limitations. Therefore, current practice often involves a combination of traditional analog approaches and digital denture treatment.

### 6.1 Analog impressions and digital fabrication

**Fig. 5:**
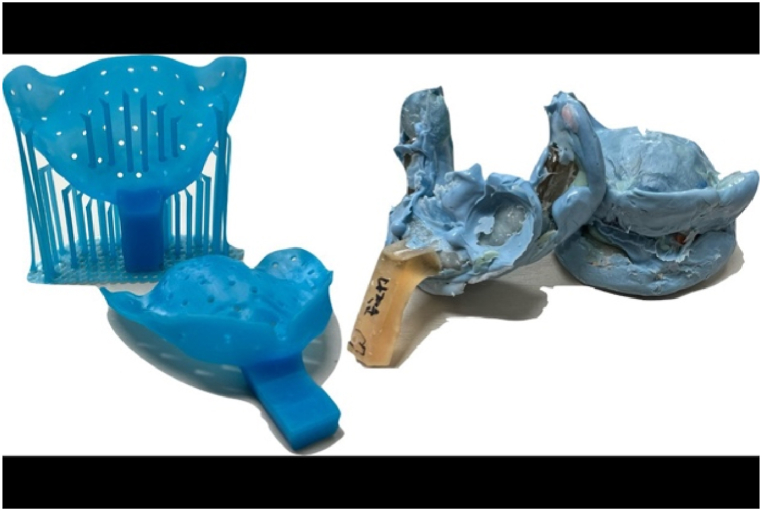
3D printing custom tray. Compared to traditional analog methods, adjusting the thickness and length of the tray is more convenient, and since the tray is fabricated with precise dimensions, it is highly advantageous for conventional impression taking.

The most common hybrid approach is to take impressions using conventional Vinyl Polysiloxane (VPS) materials and then digitize the information by scanning the impressions for subsequent digital fabrication.As previously discussed, capturing patient information for edentulous cases using intraoral scanner (IOS) is quite challenging. Therefore, dentists who are accustomed to traditional impression methods can provide digital dentures to their patients using this approach. A slight variation of this method involves digitalizing the fabrication of individual trays for impressions (Fig. 5). Rough patient information is obtained through scanning or alginate impressions, and individual trays are designed using CAD and fabricated through printing or milling. This eliminates the process of creating stone models and enables the quick production of multiple trays with uniform thickness. The limitations of intraoral scanning can be sufficiently overcome using such methods.

Another approach is to initially create a try-in denture through intraoral scanning and CAD. The try-in denture is then verified in the patient's mouth, and it is used as an individual tray for a closed-mouth technique with VPS wash impression [33]. The nearly completed denture can serve as an excellent individual tray, allowing the capture of anatomical structures that are difficult to achieve otherwise. The obtained impression is scanned together with the try-in denture outside the mouth, and these data are used to provide the patient with the final denture.

The methods described above can be selectively applied depending on the situation. The key is to incorporate traditional impression methods to overcome the difficulties of intraoral scanning. If the current limitations of intraoral scanning persist, this approach is expected to be widely used by dentists in the future.

### 6.2 Digital fabrication with analog materials

For those who wish to fabricate using digital methods but still utilize conventional materials, the following approaches can be employed. The first is to apply a library of existing teeth during the CAD process and then complete the base through printing or milling, followed by attaching the conventional teeth [46]. However, since the teeth may not fit precisely, it is recommended to create a jig that fixes all the teeth in their proper positions.

The second method involves creating a flask with a negative mold based on the digital denture file created through CAD [96]. Again, a tooth library based on existing teeth should be selected during the CAD process. The negative flask file is produced through printing or milling, and the conventional teeth are inserted. The traditional base PMMA resin, which is a mixture of liquid and powder, is then injected, and self-curing is performed. Depending on the type of flask, heat-cured resin may also be used; however, realistically producing a flask that can withstand high temperatures is challenging. Nevertheless, even if the use of heat-cured resin is difficult, excellent dentures can be fabricated using conventional materials. Although impractical, if a metal flask were to be produced, it would be possible to fabricate dentures in exactly the same manner as traditional methods.

### 6.3 Discussion on digital and analog collaboration

In a broad sense, the main categories of incorporating analog methods into digital denture fabrication are the use of conventional impression techniques and traditional materials. At first glance, these approaches may seem to undermine the advantages of digital treatment. In fact, if patient information is acquired using conventional methods, dentists may not perceive significant benefits in terms of treatment. The use of traditional materials implies additional manual work, which requires extra effort from dentists or dental technicians. Nevertheless, collaborated digital denture protocols offer various advantages. Above all, the unchanged digital denture data can be utilized permanently without time and space constraints. This work can be performed on a computer, even in locations physically distant from the dental office or patient. Moreover, if a patient loses or fractures his or her denture, a new one can be immediately produced using the existing data. Another significant advantage is the ability to fabricate dentures with relatively uniform quality regardless of the skill level of the operator. Therefore, it is currently desirable to provide treatment to patients in a way that maximizes the advantages of these collaboration methods. Most importantly, the focus should be on quickly providing patients with high-quality treatment at a reasonable cost.

## 7 Conclusion

Based on the abovementioned paper, the following points summarize the current state of digital complete denture treatment:

1) Since the introduction of CAD/CAM in dental prosthesis fabrication around 1980, digital complete denture technology was attempted approximately 10 years later and has evolved in various ways until now.
2) Compared with the established analog methods, digital denture protocols can be composed in diverse ways and vary according to the operator's preferences, treatment environment, and circumstances.
3) The fabrication methods for digital dentures are broadly categorized into milling and 3D printing; both of these techniques are undergoing continuous advancements.
4) While there are clear limitations to digitalized denture treatment methods at present, attempts are being made to overcome them.
5) The limitations of digital dentures can be largely overcome by incorporating traditional analog methods.

The key challenges in digitalized dental treatment are "full mouth reconstruction," "edentulism," and "patient information acquisition." As these issues are resolved one by one, it is believed that a significant proportion of dental treatments will be digitally transformed in the future, enabling the provision of higher-quality treatment to patients.

## Ethics statements

The facial scanning images used in this study were obtained with written informed consent from the patient for publication. Patient privacy was protected by masking identifiable features in the images. The study was exempt from IRB review as it involves minimal risk and uses completely de-identified patient data.

## Data and code availability statement

No new data was generated for the research described in the article.

## Declaration of competing interest

The authors declare that they have no known competing financial interests or personal relationships that could have appeared to influence the work reported in this paper.

## References

[1] Swoope C.C. (1974). Complete denture prosthodontics--1998. J. Prosthet. Dent.

[2] Kaplan R.L. (1963). Concepts of occlusion: gnathology as a basis for a concept of occlusion. Dent. Clin..

[3] Türp J.C., Greene C.S., Strub J.R. (2008). Dental occlusion: a critical reflection on past, present and future concepts. J. Oral Rehabil..

[4] Woelfel J.B. (1977). Processing complete dentures. Dent. Clin..

[5] Fayz F., Eslami A. (1988). Determination of occlusal vertical dimension: a literature review. J. Prosthet. Dent.

[6] Villias A., Karkazis H., Yannikakis S., Polychronakis N., Zoidis P. (2021). Current status of digital complete dentures technology. Prosthesis.

[7] Bidra A.S., Taylor T.D., Agar J.R. (2013). Computer-aided technology for fabricating complete dentures: systematic review of historical background, current status, and future perspectives. J. Prosthet. Dent.

[8] Keshvad A., Winstanley R.B., Hooshmand T. (2000). Intercondylar width as a guide to setting up complete denture teeth. J. Oral Rehabil..

[9] Woelfel J.B. (1977). Processing complete dentures. Dent. Clin..

[10] Duncan J.P., Taylor T.D. (2004). Simplified complete dentures. Dent. Clin..

[11] Duret F. (1993). The practical dental CAD/CAM in 1993. J. Can. Dent. Assoc..

[12] Bohner L.O.L., De Luca Canto G., Marció B.S., Laganá D.C., Sesma N., Tortamano Neto P. (2016). CEREC chairside system to register and design the occlusion in restorative dentistry: a systematic literature review. J. Esthetic Restor. Dent..

[13] Maeda Y., Minoura M., Tsutsumi S., Okada M., Nokubi T. (1994). A CAD/CAM system for removable denture. Part I: fabrication of complete dentures. Int. J. Prosthodont. (IJP).

[14] Kawahata N., Ono H., Nishi Y., Hamano T., Nagaoka E. (1997). Trial of duplication procedure for complete dentures by CAD/CAM. J. Oral Rehabil..

[15] Anadioti E., Musharbash L., Blatz M.B., Papavasiliou G., Kamposiora P. (2020). 3D printed complete removable dental prostheses: a narrative review. BMC Oral Health.

[16] Park J.H., Cho I.H., Shin S.Y., Choi Y.S. (2015). The treatment of an edentulous patient with DENTCA $^{TM} $ CAD/CAM Denture. J Korean Acad Prosthodont.

[17] Paryag A., Rafeek R., Meighan A. (2018). Using a simple chair-side copy denture technique in the AvaDent digital denture process: a case report and review. Open J. Stomatol..

[18] Andreescu C.F., Radu V., Dinu L., Brumaru M., Stoica M.C. (2018). Evaluation of different materials used for fabrication of complete digital denture. Mater. Plast..

[19] Ali I.E., Alammari M.R., Aref N.S. (2023). Applications and performance of artificial intelligence models in removable prosthodontics: a literature review. J Prosthodont Res.

[20] Alshadidi A.A.F., Marghalani A., Moaleem M.M.A., Abdullah M., Ali H.S., Shahul S. (2023). Investigation on the application of artificial intelligence in prosthodontics. Appl. Sci..

[21] Lee S., Hong S.J., Paek J., Pae A., Kwon K.R. (2019). Comparing accuracy of denture bases fabricated by injection molding, CAD/CAM milling, and rapid prototyping method. J Adv Prosthodont.

[22] Negreiros W.M., Hamilton A., Gallucci G.O. (2022). A completely digital workflow for the transition from a failed dentition to interim complete-arch fixed implant-supported prostheses: a clinical report. J. Prosthet. Dent.

[23] Coachman C., Sesma N., Blatz M.B. (2021). The complete digital workflow in interdisciplinary dentistry. Int J Esthet Dent..

[24] Salagare S., Prasad R. (2020). An overview of internet of dental things: new frontier in advanced dentistry. Wireless Pers. Commun..

[25] Devan M.M. (2005). Basic principles in impression making. 1952. J. Prosthet. Dent.

[26] Gómez-Polo M., Immorlano M.G., Cascos-Sánchez R., Ortega R., Barmak A.B., Kois J.C. (2023). Influence of the dental arch and number of cutting-off and rescanning mesh holes on the accuracy of implant scans in partially edentulous situations. J. Dent..

[27] Wang X., Zhang F., Ma D., Ye X., Zheng X., Ren R. (2024). Evaluation of the accuracy of seven intraoral scanners for the full dentate and partially edentulous complete-arch mandibular casts: an in vitro comparison. Heliyon.

[28] Dupagne L., Tapie L., Lebon N., Mawussi B. (2022). Comparison of the acquisition accuracy and digitizing noise of 9 intraoral and extraoral scanners: an objective method. J. Prosthet. Dent.

[29] Schmalzl J., Keskeny G.Á., Hermann P., Pál A., Géczi Z., Borbély J. (2024). Evaluating the influence of palate scanning on the accuracy of complete-arch digital impressions-An in vitro study. J. Dent..

[30] Parvizi A., Lindquist T., Schneider R., Williamson D., Boyer D., Dawson D.V. (2004). Comparison of the dimensional accuracy of injection-molded denture base materials to that of conventional pressure-pack acrylic resin. J. Prosthodont..

[31] Jivănescu A., Bratu E.A., Bolos A., Marsavina L., Pop D.M., Negrutiu M.L. (2021). Is there a significant difference in accuracy of four intraoral scanners for short-span fixed dental prosthesis? A comparative in vitro study. Appl. Sci..

[32] Rao S., Chowdhary R., Mahoorkar S. (2010). A systematic review of impression technique for conventional complete denture. J. Indian Prosthodont. Soc..

[33] Lo Russo L., Guida L., Ronsivalle V., Ercoli C. (2024). Digital denture with mucostatic base and functional borders: a cast-free digital technique. J. Prosthodont..

[34] Lo Russo L., Salamini A., Troiano G., Guida L. (2021). Digital dentures: a protocol based on intraoral scans. J. Prosthet. Dent.

[35] Kattadiyil M.T., Jekki R., Goodacre C.J., Baba N.Z. (2015). Comparison of treatment outcomes in digital and conventional complete removable dental prosthesis fabrications in a predoctoral setting. J. Prosthet. Dent.

[36] Fayz F., Eslami A. (1988). Determination of occlusal vertical dimension: a literature review. J. Prosthet. Dent.

[37] Ismail Y.H., George W.A. (1968). The consistency of the swallowing technique in determining occlusal vertical relation in edentulous patients. J. Prosthet. Dent.

[38] Weinberg L.A. (1982). Vertical dimension: a research and clinical analysis. J. Prosthet. Dent.

[39] Alehaideb A., Lin W.S., Levon J.A., Chu T.G., Yang C.C. (2023). Accuracy of digital duplication scanning methods for complete dentures. J. Prosthodont..

[40] Troncoso-Pazos J., Matamala P., Jusari M.F., Risco K., Aguilera F.R., Aravena P.C. (2023). Position of digitally guided implants in completely edentulous maxillae by using a modified double-scan and overlap of three digital surface protocol. J. Prosthet. Dent.

[41] Wada M., Mameno T., Tsujioka Y., Yamashita M., Ikebe K. (2022). Effective utilization of digital technology in complete denture fabrication. J. Oral Sci..

[42] Zhang S., Lin Y., Chen W., Chen J. (2023). A fully digital 1-day technique for fabricating complete dentures based on an existing denture. J. Prosthet. Dent.

[43] Wehner P.J., Hickey J.C., Boucher C.O. (1967). Selection of artificial teeth. J. Prosthet. Dent.

[44] Lombardi R.E. (1973). The principles of visual perception and their clinical application to denture esthetics. J. Prosthet. Dent.

[45] Li W., Chen H., Wang Y., Xie Q., Sun Y. (2022). Digital determination and recording of edentulous maxillomandibular relationship using a jaw movement tracking system. J. Prosthodont..

[46] Infante L., Yilmaz B., McGlumphy E., Finger I. (2014). Fabricating complete dentures with CAD/CAM technology. J. Prosthet. Dent.

[47] Jacobson T.E., Krol A.J. (1983). A contemporary review of the factors involved in complete denture retention, stability, and support. Part I: retention. J. Prosthet. Dent.

[48] Jacobson T.E., Krol A.J. (1983). A contemporary review of the factors involved in complete denture retention, stability, and support. Part II: stability. J. Prosthet. Dent.

[49] Jacobson T.E., Krol A.J. (1983). A contemporary review of the factors involved in complete dentures. Part III: support. J. Prosthet. Dent.

[50] Lee S.Y., Kim H., Lee D., Park C. (2021). Superimposition of a cone beam computed tomography (CBCT) scan and a photograph: a dental technique. J. Prosthet. Dent.

[51] Satin S.R., Goodacre B.J., Masri R. (2024). Comparing the accuracy of occlusal vertical dimension transfer in CAD-CAM dentures. J. Prosthodont..

[52] Fekri L.K., Abdelaziz M.S. (2023). Digital duplication of maxillary complete denture: a dental technique. J. Esthetic Restor. Dent..

[53] Revilla-León M., Zeitler J.M., Kois J.C. (2024). An overview of the different digital facebow methods for transferring the maxillary cast into the virtual articulator. J. Esthetic Restor. Dent..

[54] Geiger B., Mehl A. (2024). A novel algorithmic approach for automatic virtual articulation to avoid dynamic interferences in dental restoration designs. Int. J. Comput. Dent..

[55] Kanazawa M., Inokoshi M., Minakuchi S., Ohbayashi N. (2011). Trial of a CAD/CAM system for fabricating complete dentures. Dent. Mater. J..

[56] Kashiwazaki K., Komagamine Y., Namano S., Park J.M., Iwaki M., Minakuchi S. (2023). Prediction accuracy of incisal points in determining occlusal plane of digital complete dentures. J Adv Prosthodont.

[57] Fang J.H., An X., Jeong S.M., Choi B.H. (2018). Digital intraoral scanning technique for edentulous jaws. J. Prosthet. Dent.

[58] Yang J.W., Zhu Y.J., Afrashtehfar K.I., Zhao Y.J., Wang Y. (2023). Enhanced lip-contour during facial scan for 3D DSD and implant planning: a blue screen approach. J. Oral Implantol..

[59] Jankelson B. (1962). Adjustment of dentures at time of insertion and alterations to compensate for tissue change. J. Am. Dent. Assoc..

[60] Cai H., Xu X., Lu X., Zhao M., Jia Q., Jiang H.B. (2023). Dental materials applied to 3D and 4D printing technologies: a review. Polymers.

[61] AlRumaih H.S. (2021). Clinical applications of intraoral scanning in removable prosthodontics: a literature review. J. Prosthodont..

[62] Pilecco R.O., Machry R.V., Baldi A., Tribst J.P.M., Sarkis-Onofre R., Valandro L.F. (2024). Influence of CAD-CAM milling strategies on the outcome of indirect restorations: a scoping review. J. Prosthet. Dent.

[63] Zandinejad A., Floriani F., Lin W.S., Naimi-Akbar A. (2024). Clinical outcomes of milled, 3D-printed, and conventional complete dentures in edentulous patients: a systematic review and meta-analysis. J. Prosthodont..

[64] Oyar P., Ulusoy M. (2024). Effect of milling procedures in CAD-CAM systems on the color changes of CAD-CAM polymethyl methacrylate resin material as interim material. BMC Oral Health.

[65] Srinivasan M., Kamnoedboon P., McKenna G., Angst L., Schimmel M., Özcan M. (2021). CAD-CAM removable complete dentures: a systematic review and meta-analysis of trueness of fit, biocompatibility, mechanical properties, surface characteristics, color stability, time-cost analysis, clinical and patient-reported outcomes. J. Dent..

[66] Jung S., Park C., Yang H.S., Lim H.P., Yun K.D., Ying Z. (2019). Comparison of different impression techniques for edentulous jaws using three-dimensional analysis. J Adv Prosthodont.

[67] Hwang H.J., Lee S.J., Park E.J., Yoon H.I. (2019). Assessment of the trueness and tissue surface adaptation of CAD-CAM maxillary denture bases manufactured using digital light processing. J. Prosthet. Dent.

[68] Kessler A., Hickel R., Reymus M. (2020). 3D printing in dentistry-state of the art. Operat. Dent..

[69] Hytham A., Osman R.B. (2024). The journey of additive manufacturing in prosthodontics from the early dawn till the current state of art. A narrative review. Int. J. Prosthodont. (IJP).

[70] Zhan X., Cao L., Xiang D., Tang H., Xia D., Lin H. (2024). [Effect of printing orientation on physical and mechanical properties of 3D printing prosthodontic base resin materials]. Beijing Da Xue Xue Bao Yi Xue Ban.

[71] Perlea P., Stefanescu C., Dalaban M.G., Petre A.E. (2024). Experimental study on dimensional variations of 3D printed dental models based on printing orientation. Clin Case Rep.

[72] Goodacre B.J. (2024). 3D printing of complete dentures: a narrative review. Int. J. Prosthodont. (IJP).

[73] Han D.S., Kim R., Hyun H.K., Yoon H.I., Jeong H.R., Park C. (2024). The impact of oxygen concentration on the postcuring of 3D-printed dental resin. Int. J. Prosthodont. (IJP).

[74] Song G., Son J.W., Jang J.H., Choi S.H., Jang W.H., Lee B.N. (2021). Comparing volumetric and biological aspects of 3D-printed interim restorations under various post-curing modes. J Adv Prosthodont.

[75] Oh R., Lim J.H., Lee C.G., Lee K.W., Kim S.Y., Kim J.E. (2023). Effects of washing solution temperature on the biocompatibility and mechanical properties of 3D-Printed dental resin material. J. Mech. Behav. Biomed. Mater..

[76] Bidra A.S., Taylor T.D., Agar J.R. (2013). Computer-aided technology for fabricating complete dentures: systematic review of historical background, current status, and future perspectives. J. Prosthet. Dent.

[77] Kattadiyil M.T., Jekki R., Goodacre C.J., Baba N.Z. (2015). Comparison of treatment outcomes in digital and conventional complete removable dental prosthesis fabrications in a predoctoral setting. J. Prosthet. Dent.

[78] Kattadiyil M.T., Jekki R., Goodacre C.J., Baba N.Z. (2015). Comparison of treatment outcomes in digital and conventional complete removable dental prosthesis fabrications in a predoctoral setting. J. Prosthet. Dent.

[79] Vitai V., Németh A., Sólyom E., Czumbel L.M., Szabó B., Fazekas R. (2023). Evaluation of the accuracy of intraoral scanners for complete-arch scanning: a systematic review and network meta-analysis. J. Dent..

[80] Jin C.X., Lou M.W., Cai X.J., Li M.X., Huang Q.C., Niu L.N. (2024). [A two-dimensional photographic and three-dimensional digital dental model comparative analysis in maxillary anterior teeth]. Zhonghua Kou Qiang Yi Xue Za Zhi.

[81] Aguirre B.C., Chen J.H., Kontogiorgos E.D., Murchison D.F., Nagy W.W. (2020). Flexural strength of denture base acrylic resins processed by conventional and CAD-CAM methods. J. Prosthet. Dent.

[82] Arslan M., Murat S., Alp G., Zaimoglu A. (2018). Evaluation of flexural strength and surface properties of prepolymerized CAD/CAM PMMA-based polymers used for digital 3D complete dentures. Int. J. Comput. Dent..

[83] Takaichi A., Fueki K., Murakami N., Ueno T., Inamochi Y., Wada J., Arai Y., Wakabayashi N. (2022). A systematic review of digital removable partial dentures. Part II: CAD/CAM framework, artificial teeth, and denture base. J Prosthodont Res.

[84] Lawson N.C., Bansal R., Burgess J.O. (2016). Wear, strength, modulus and hardness of CAD/CAM restorative materials. Dent. Mater..

[85] Mubaraki M.Q., Moaleem M.M.A., Alzahrani A.H., Shariff M., Alqahtani S.M., Porwal A. (2022). Assessment of conventionally and digitally fabricated complete dentures: a comprehensive review. Materials.

[86] Muslimah D.F., Hasegawa Y., Antonin T., Richard F., Hosaka K. (2024). Composite injection technique with a digital workflow: a pragmatic approach for a protruding central incisor restoration. Cureus.

[87] Abreu A., Londono J., Torosian A., Yu J., Levy-Bercowski D. (2021). Aesthetic concepts and interdisciplinary approach in a patient with bilateral cleft lip and palate and missing premaxilla: a case report. Cleft Palate Craniofac J.

[88] Reddy E. Jayakiran, Venkatachalapathi N., Pandu Rangadu V. (2018). "Development of an approach for knowledge-based system for CAD modelling. Mater. Today.

[89] Jeong Y.G., Lee W.S., Lee K.B. (2018). Accuracy evaluation of dental models manufactured by CAD/CAM milling method and 3D printing method. J Adv Prosthodont.

[90] Bilgin M.S., Baytaroğlu E.N., Erdem A., Dilber E. (2016). A review of computer-aided design/computer-aided manufacture techniques for removable denture fabrication. Eur. J. Dermatol..

[91] Wang C., Shi Y.F., Xie P.J., Wu J.H. (2021). Accuracy of digital complete dentures: a systematic review of in vitro studies. J. Prosthet. Dent.

[92] Namano S., Kanazawa M., Katheng A., Trang B.N.H., Hada T., Komagamine Y. (2024). Effect of support structures on the trueness and precision of 3D printing dentures: an in vitro study. J Prosthodont Res.

[93] Balhaddad A.A., Alqahtani F.Y., Alshammari A.F., AlQahtani H.S., Alqahtani R.A., Abdulmajeed J. (2023). Three-dimensional (3D) printing in dental practice: applications, areas of interest, and level of evidence. Clin. Oral Invest..

[94] Kim H., Lee D., Lee S.Y., Yang H., Park S.W., Lim H.P. (2020). Denture flask fabrication using fused deposition modeling three-dimensional printing. J Prosthodont Res.

[95] Sulaiman T.A. (2020). Materials in digital dentistry-A review. J. Esthetic Restor. Dent..

[96] Piao X.Y., Jeon J., Shim J.S., Park J.M. (2022). A digital workflow for the fabrication of a milled removable partial denture. Int. J. Environ. Res. Publ. Health.

